# Supplementary figures and images for: Chromatin modifiers and recombination factors promote a telomere fold-back structure, that is lost during replicative senescence
Source: PLoS Genet. 2020 Dec 28;16(12):e1008603. doi: 10.1371/journal.pgen.1008603 (PMC7793543; doi:10.1371/journal.pgen.1008603)

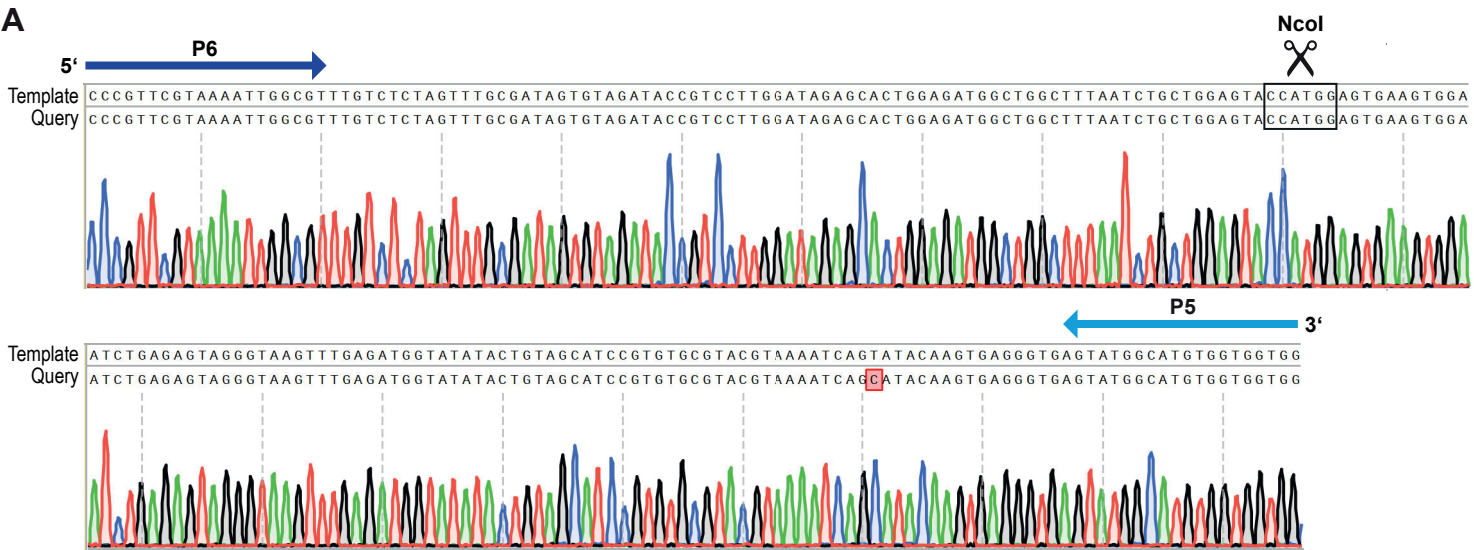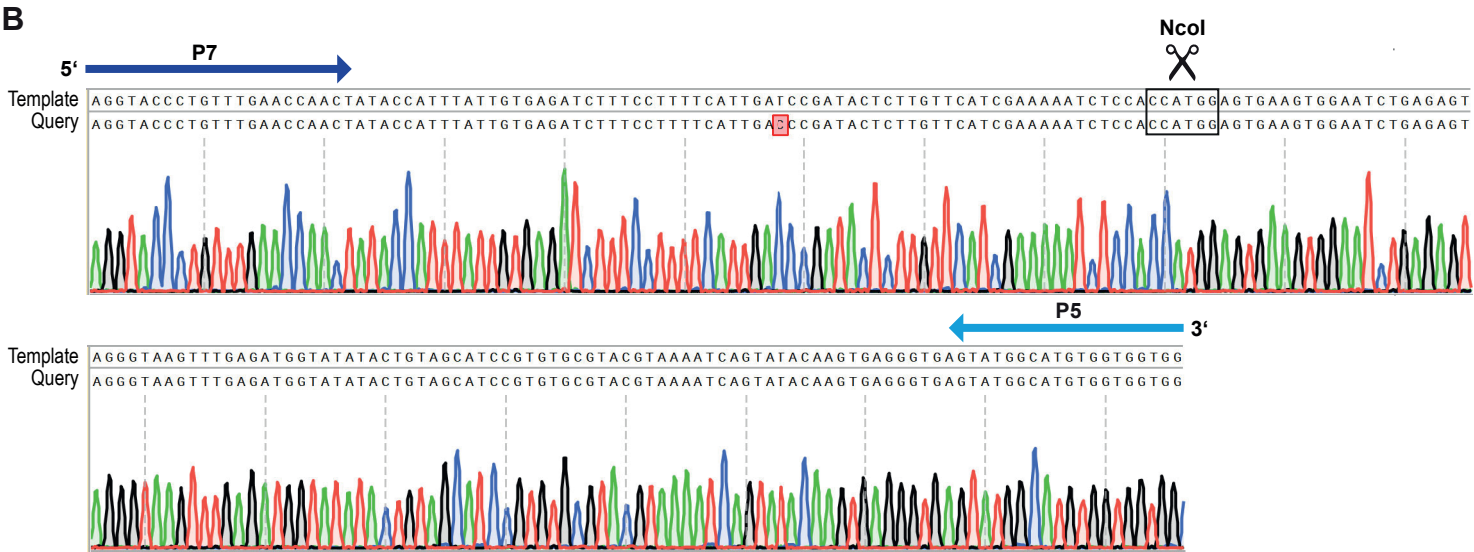

Supplement: S1 Fig — A. Sanger sequencing chromatogram of the Telo-3C qPCR product using the primers P5 and P6. The qPCR product was subcloned for sequencing. The sequence corresponds to the template sequence from telomere 1L. One mismatch is indicated as a red square. B. Sanger sequencing chromatogram of the Telo-3C qPCR product using the primers P5 and P7. The qPCR product was subcloned for sequencing. The sequence corresponds to the template sequence from telomere 1L. One mismatch is indicated as a red square. (PDF) [file pgen.1008603.s001.pdf]

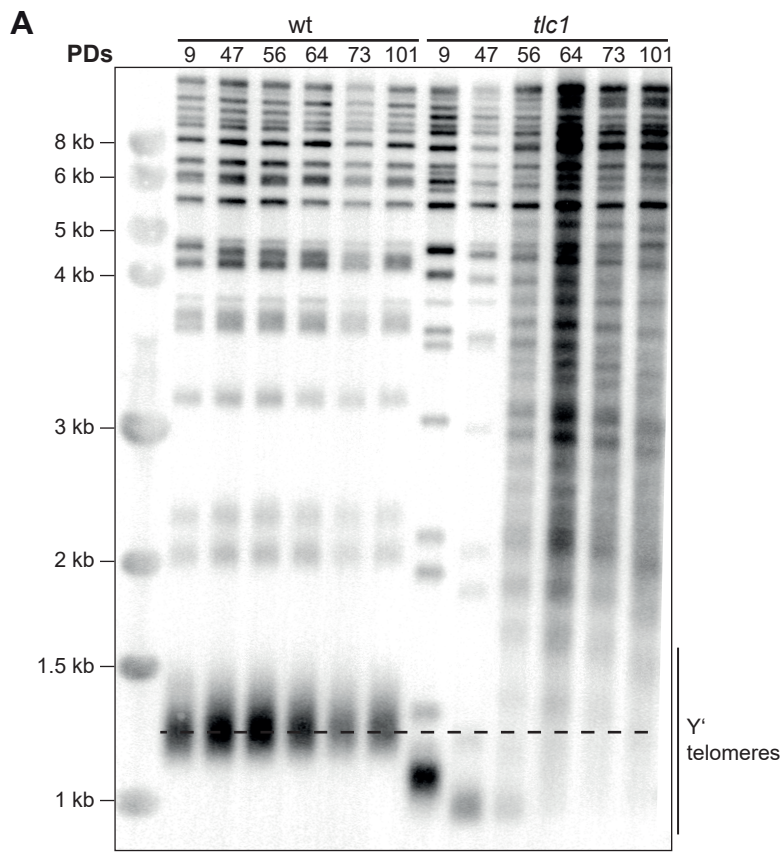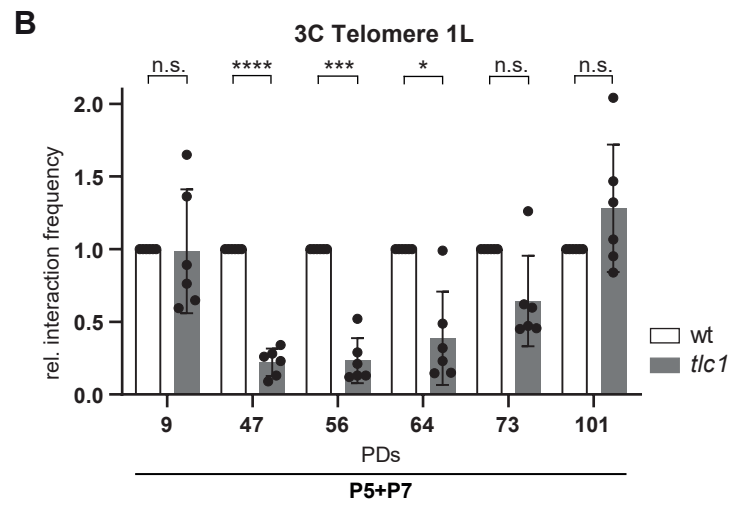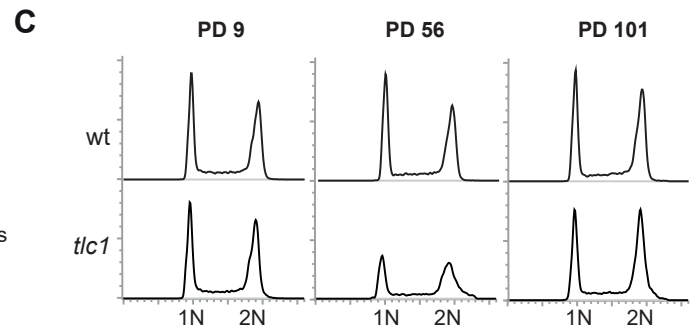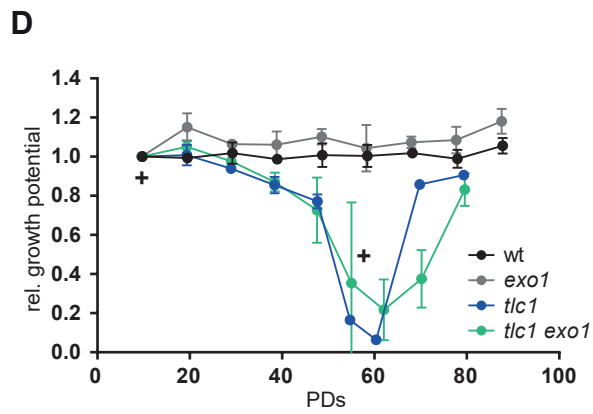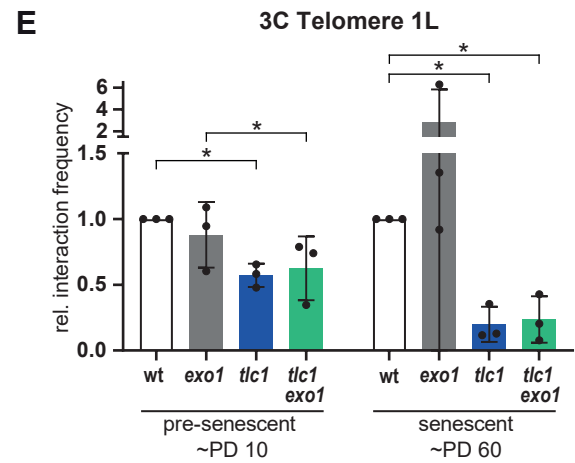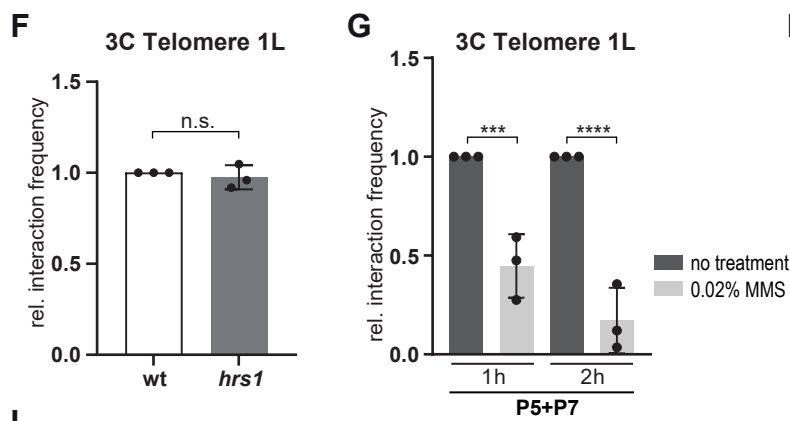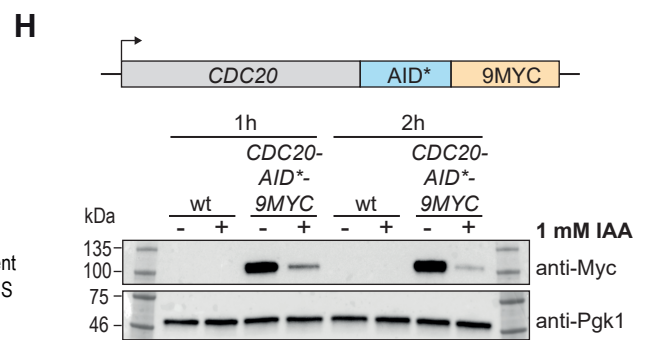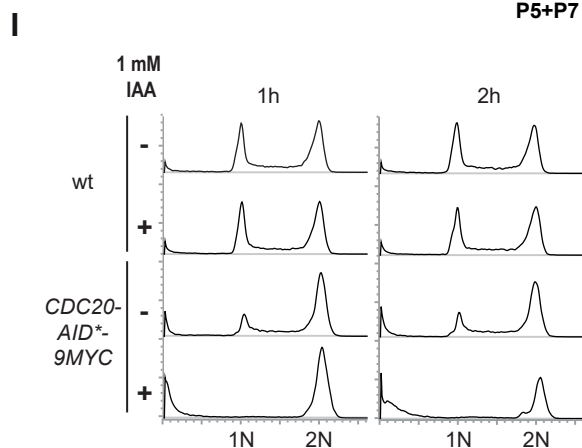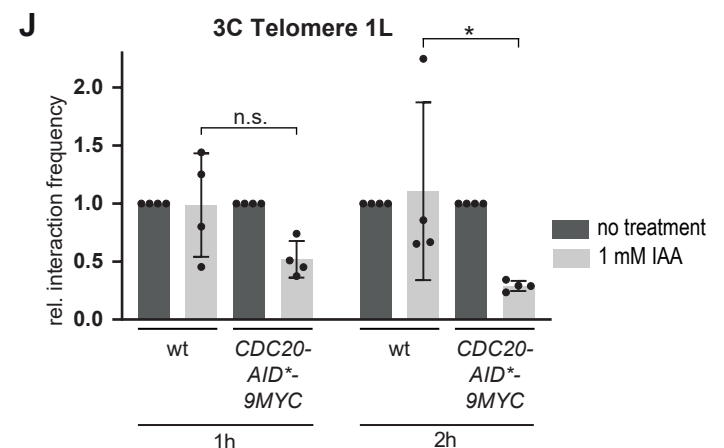

Supplement: S2 Fig — A. Southern blot for all telomeres on XhoI-digested genomic DNA using a radio-labeled (TG)n-repeat containing vector fragment as a probe. One representative sample for each genotype is shown (dashed grey line in Fig 2A). The dashed black line indicates wt telomere length. B. Telo-3C analysis during the time course shown in Fig 2A. The relative interaction frequencies between primers P5 and P7 (Fig 1B) are shown. All interaction frequencies were normalized to a control qPCR product. Relative interaction frequencies were calculated by setting the normalized interaction frequency of the wt of the respective day to 1. Population doubling values that were used for Telo-3C correspond to those used in the senescence curve (Fig 2A) (i.e. from 24-hour saturated cultures). Mean +/- SD of 6 different clones in 3 independent experiments. Adjusted p-values were obtained from two-way ANOVA with Sidak’s multiple comparisons test (*p ≤ 0.05, ***p ≤ 0.001, ****p ≤ 0.0001, n.s., not significant). C. Flow cytometry histograms for DNA content of wt and tlc1 cells during the time course shown in Fig 2A. One representative sample per genotype is shown. D. The relative growth potential of 3 independent cultures of each genotype (wt, exo1, tlc1, exo1 tlc1) was followed over ~90 PDs. Samples for Telo-3C were collected at the indicated time points (+). We defined PD 0 as the time the senescence curve was started in liquid media from the germinated spore colony. E. Telo-3C analysis of wt, exo1, tlc1 and exo1 tlc mutants in pre-senescent (~PD 10) and senescent (~PD 60) cells as shown in S2D Fig. The relative interaction frequencies between primers P5 and P6 are shown. All interaction frequencies were normalized to a control qPCR product. Relative interaction frequencies were calculated by setting the normalized interaction frequency of the wt of the respective day to 1. Mean +/- SD of 3 independent experiments. Adjusted p-values were obtained from two-way ANOVA with Tukey’s multiple comparison [file pgen.1008603.s002.pdf]

**A**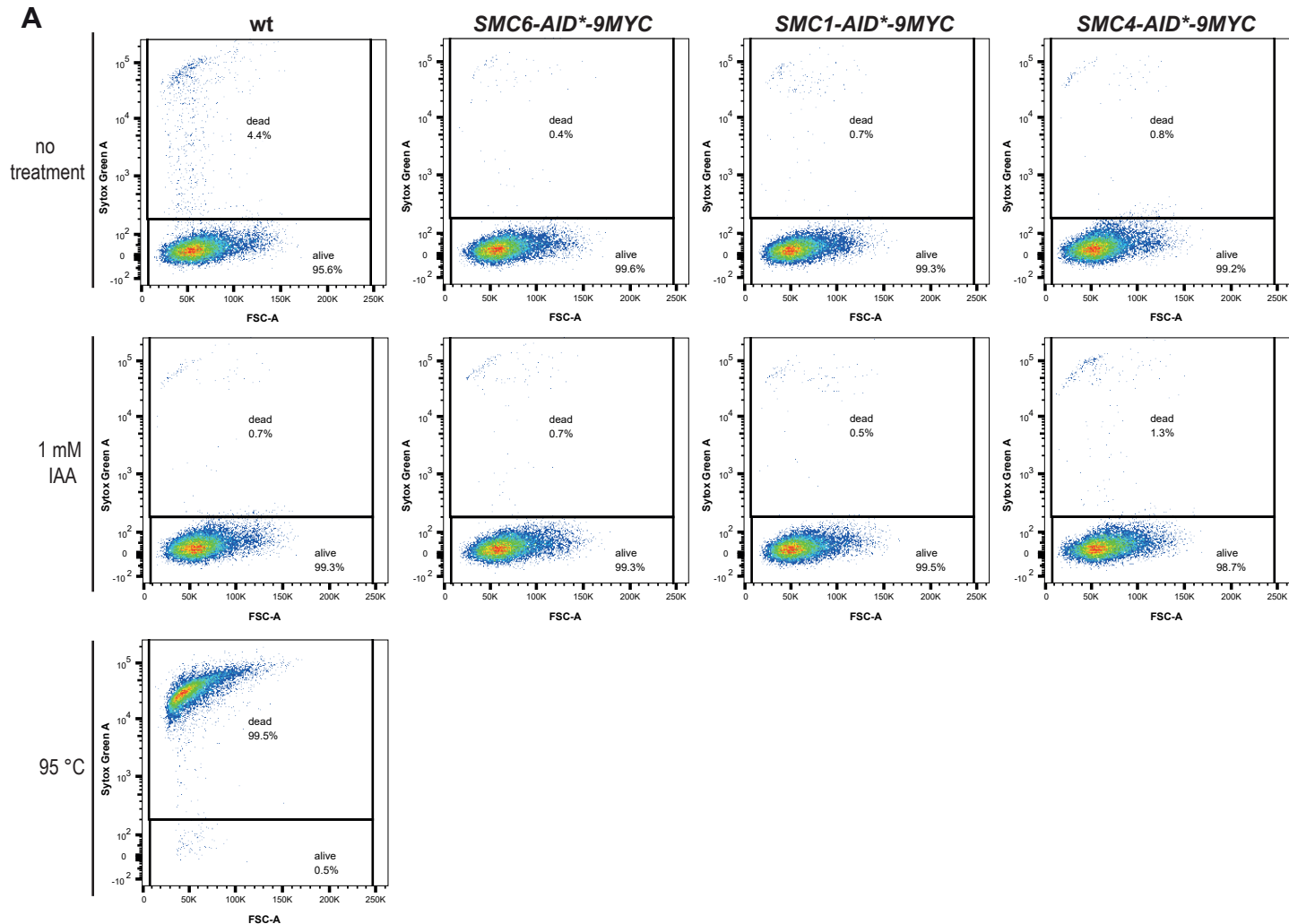**B**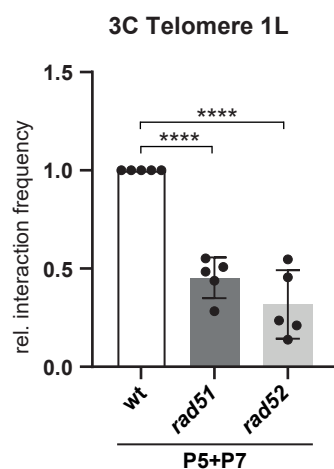**D**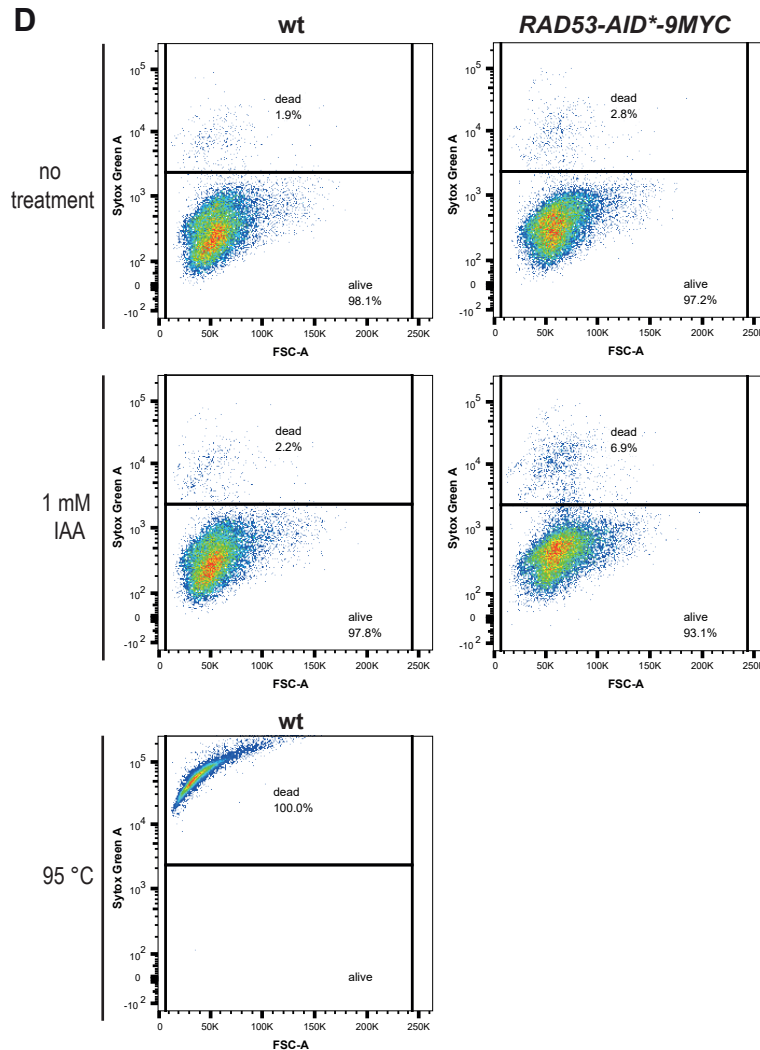**C**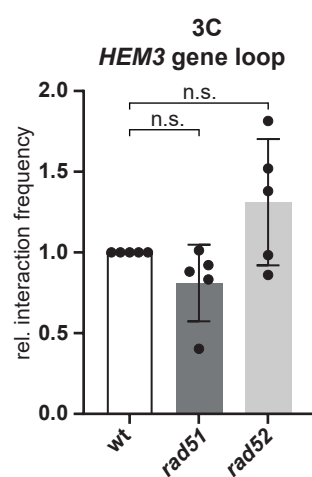

Supplement: S3 Fig — A. Flow cytometry histograms for viability using SYTOX green stain after the depletion of the SMC complex members for 1 h (treatment with 1 mM IAA). As a control for dead cells, wt cells were incubated for 15 min at 95°C. B. Telo-3C analysis of rad51 and rad52 mutants. The interaction frequencies between primers P5 and P7 are shown. All interaction frequencies were normalized to a control qPCR product. Relative interaction frequencies were calculated by setting the normalized interaction frequency of the wt samples to 1. Mean +/- SD of 5 independent experiments. Adjusted p-values were obtained from one-way ANOVA with Dunnett’s multiple comparisons test (****p ≤ 0.0001). C. 3C analysis for the HEM3 gene loop in rad51 and rad52 mutants. All interaction frequencies were normalized to a control qPCR product. Relative interaction frequencies were calculated by setting the normalized interaction frequency of the wt samples to 1. Mean +/- SD of 5 independent experiments. Adjusted p-values were obtained from one-way ANOVA with Dunnett’s multiple comparisons test (n.s., not significant). D. Flow cytometry histograms for viability using SYTOX green stain after the depletion of Rad53 for 2 h (treatment with 1 mM IAA). As a control for dead cells, wt cells were incubated for 15 min at 95°C. IAA = Indole-3 acetic acid. (PDF) [file pgen.1008603.s003.pdf]

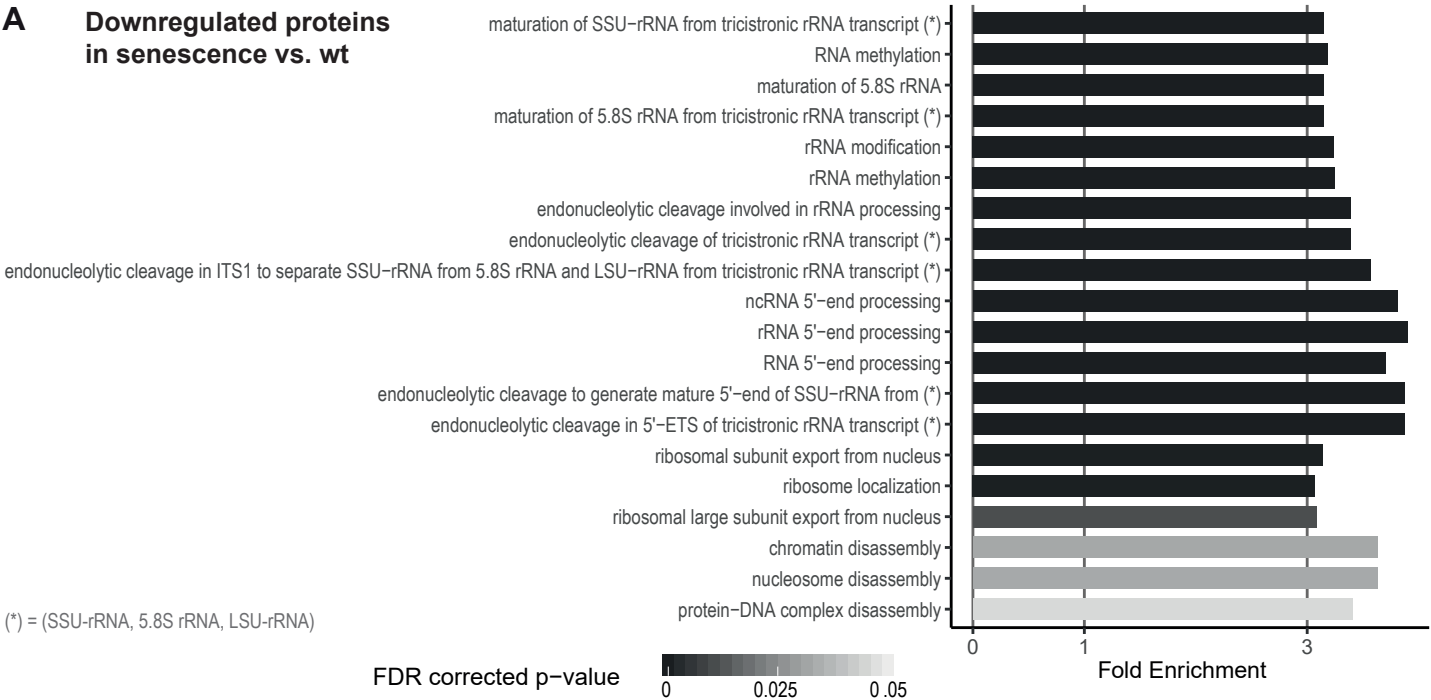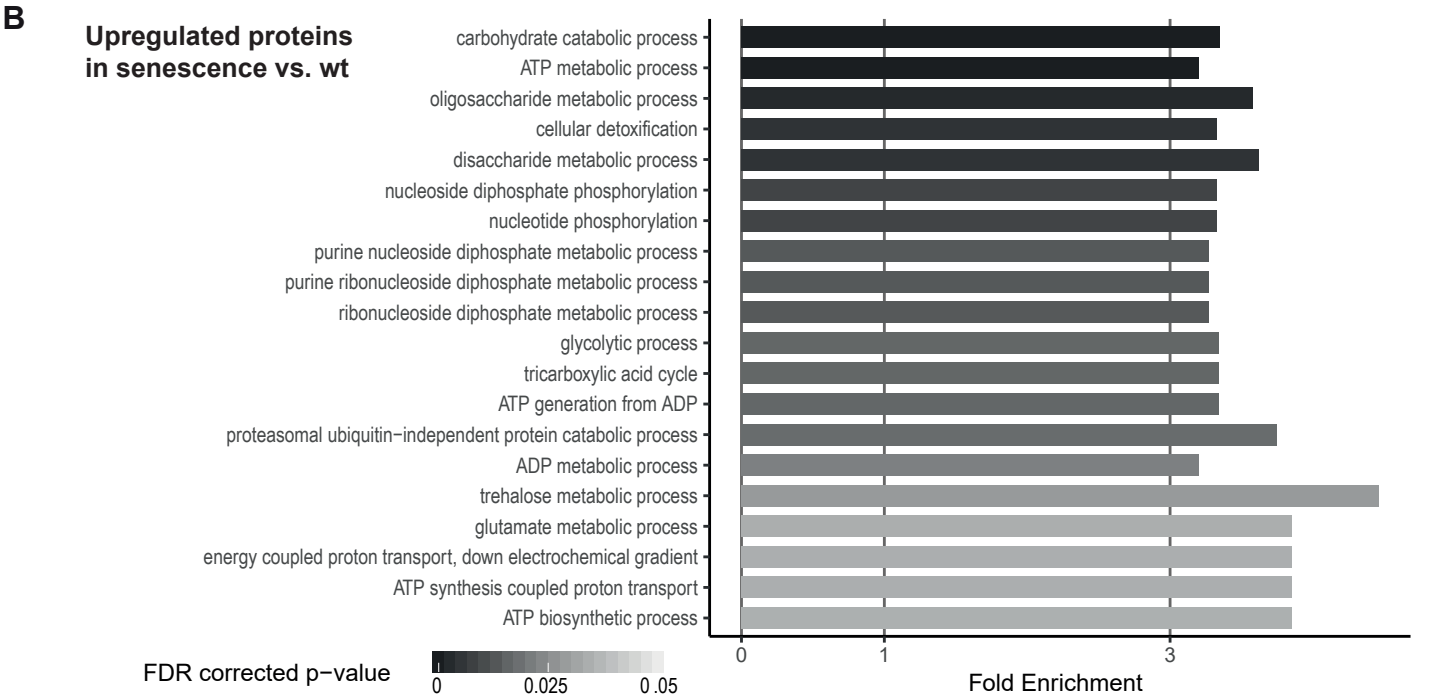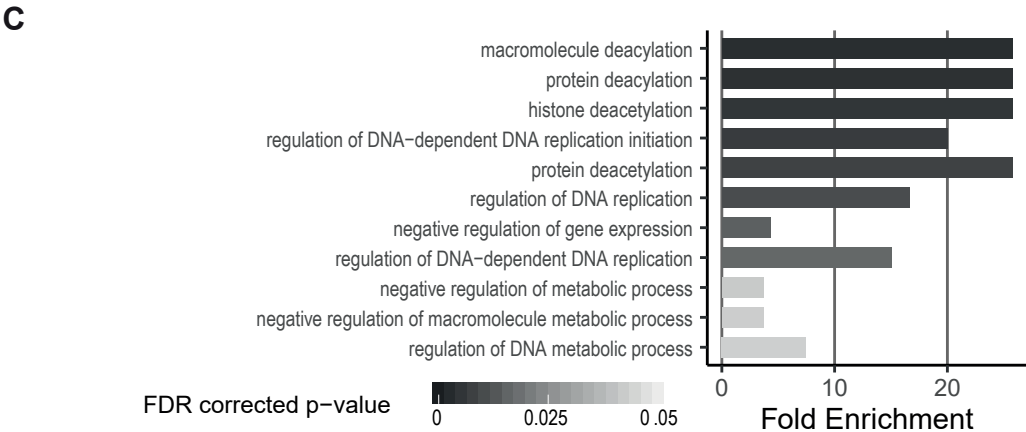

Supplement: S5 Fig — A. Top 20 gene ontology (GO) terms for biological processes of 751 proteins downregulated in senescence (tlc1) compared to wt. B. Top 20 GO terms for biological processes of 678 proteins upregulated in senescence (tlc1) compared to wt. C. Results from gene ontology analysis for molecular function of the 21 overlapping candidate genes from (Fig 4B). Shown are GO terms with p-values below 0.05. sir2 was included in the list of fold-back defective mutants. (PDF) [file pgen.1008603.s005.pdf]

**A**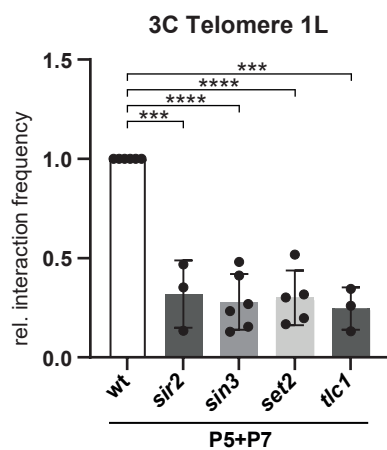**B**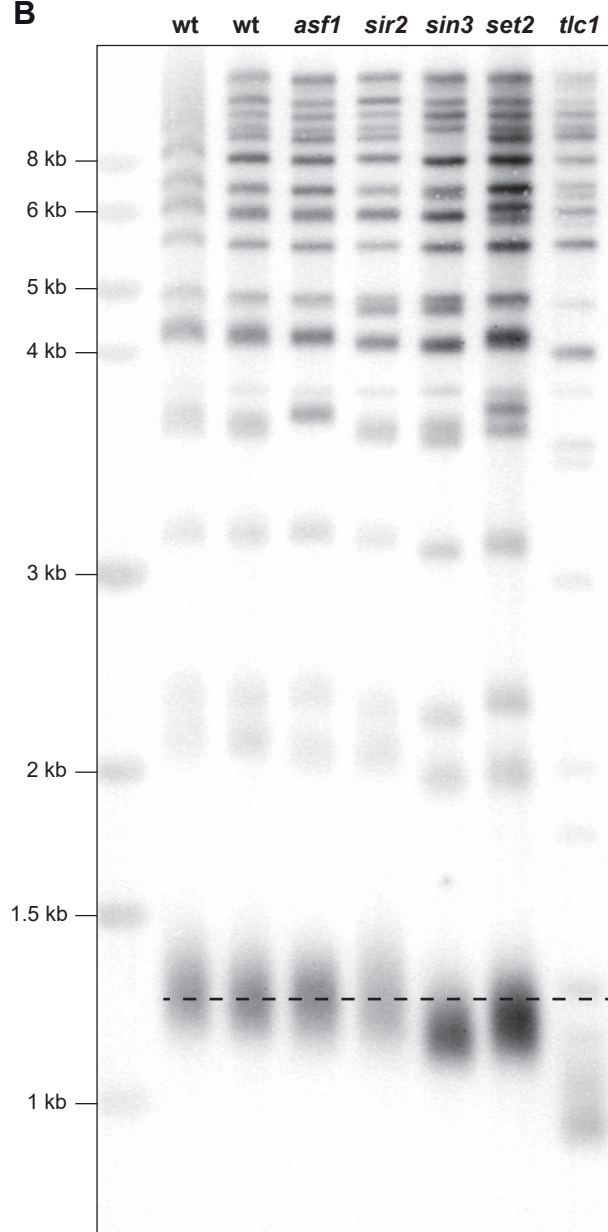**C**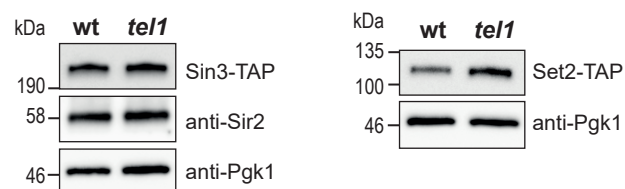**D**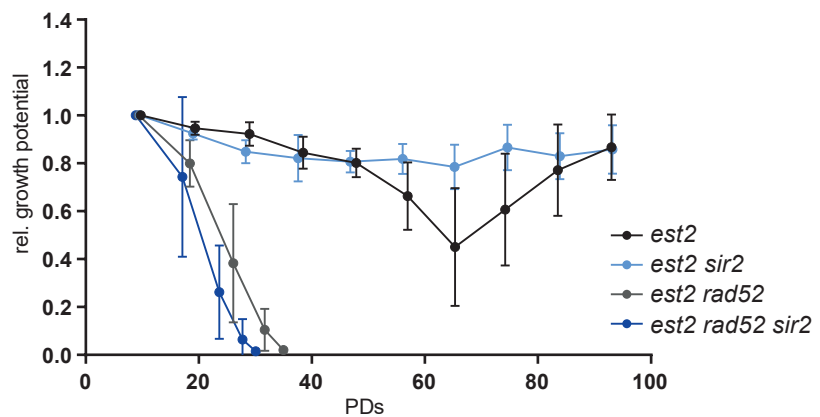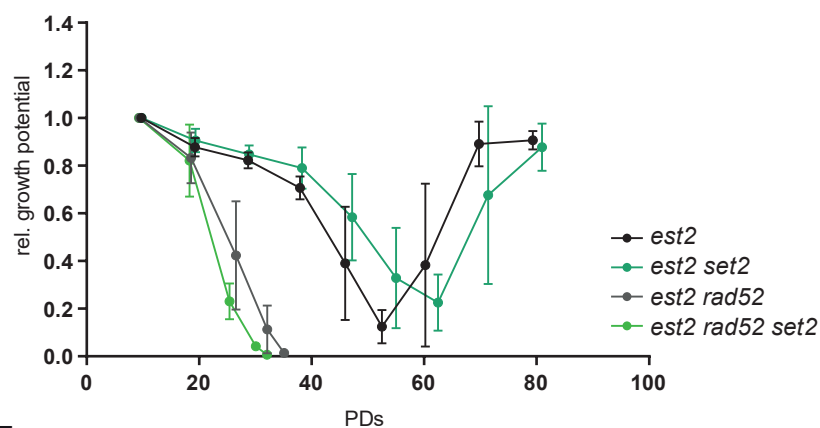**E**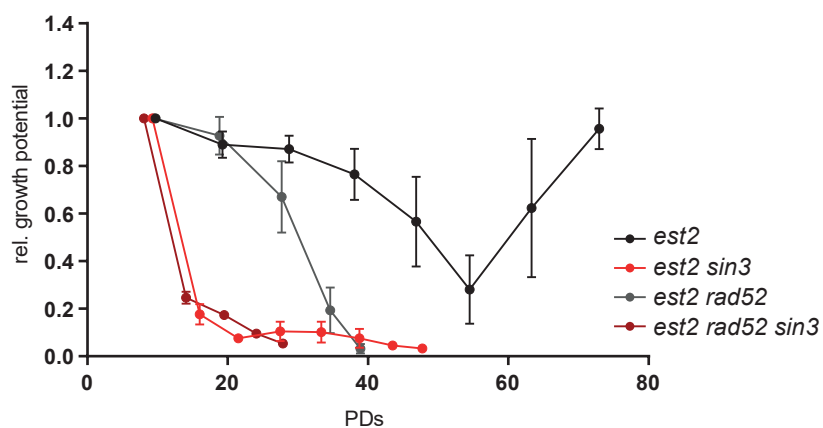

Supplement: S6 Fig — A. Telo-3C analysis of sir2, sin3, set2 and tlc1 (~PD 40) mutants. The interaction frequencies between primers P5 and P7 are shown. All interaction frequencies were normalized to a control qPCR product. Relative interaction frequencies were calculated by setting the normalized interaction frequency of the wt to 1. Mean +/- SD of 3–6 independent experiments. For sir2 and tlc1 mutants adjusted p-values were obtained from one-way ANOVA with Dunnett’s multiple comparisons test. For sin3 and set2 mutants adjusted p-values were obtained from unpaired t-tests (***p ≤ 0.001, ****p ≤ 0.0001). B. Uncropped Southern blot corresponding to Fig 4F. C. Western blot of endogenously TAP-tagged Sin3 and Set2, as well as Sir2 levels in tel1 mutants. D. The relative growth potential of 6 independent cultures was followed over ~100 PDs in mutants with the indicated genotypes. We defined PD 0 as the time the senescence curve was started in liquid media from the germinated spore colony. E. The relative growth potential of 6 independent cultures was followed over ~100 PDs in mutants with the indicated genotypes. We defined PD 0 as the time the senescence curve was started in liquid media from the germinated spore colony. PDs = population doublings. (PDF) [file pgen.1008603.s006.pdf]
