# Supplementary material for: Chromatin modifiers and recombination factors promote a telomere fold-back structure, that is lost during replicative senescence
Source: PLoS Genet. 2020 Dec 28;16(12):e1008603. doi: 10.1371/journal.pgen.1008603 (PMC7793543; doi:10.1371/journal.pgen.1008603)
Supplement: S4 Fig — List of all significantly up- or downregulated proteins in senescent tlc1 cells versus wt cells (p ≤ 0.05) with log2 fold change (blue: upregulated; red: downregulated). (PDF) [file pgen.1008603.s004.pdf]

RNR3	PUT1	CRG1	GDB1	YMR196W	HUG1	RTC3	TFS1	YGP1	YBR085C-A	GSY1	YHR138C	GPH1	ALD3	MRP49	PET10
RNR4	RNR2	BNA2	HSP26	GOR1	ATG8	GLC3	TSA2	PNC1	SOL4	EGO4	RIM4	GAD1	YMR090W	GCY1	USA1
HSP12	AAR2	TMA10	GSY2	GTT1	RNY1	GLK1	HSP31	YBR053C	ECM4	PGM2	AMS1	TMA17	TDH1	COQ3	PRX1
GSC2	FIS1	GPM2	PIC2	CYC7	HXK1	DCS2	MSC1	YNL058C	HSP42	SCO2	FMP46	TMT1	DUR1,2	MRX14	RAD51
YDR391C	YJR096W	YDL124W	STE18	GRX1	ENO1	NQM1	AIM41	YNL332W	NNR2	INH1	YOL055C	SSA4	ALD2	YJL132W	PRB1
TPS1	TRX3	QCR7	NPC2	QCR2	MCR1	OM45	APE1	SNZ1	BNA1	GND2	PBI2	PDH1	YCH1	YBR056W	NNR1
COX2	RDL1	PEP4	CAR1	ARA1	GPP2	UGA1	SLT2	SPC2	PST2	YHR033W	TSL1	RIB5	GPD1	HYR1	AIM17
LOT6	SOD2	DCS1	AYR1	SRY1	CAR2	HBN1	FRD1	UGA2	GCG1	RDI1	POR1	LSC2	MPM1	TPS2	YNL134C
SSE2	COQ9	SER3	YDR001C	ATP5	GDH2	PTP1	YJL068C	ARG4	MPT5	YNL115C	MRP8	YHR043C	RCN2	YGR127W	HPA3
GRX2	MMS2	PPZ1	CDD1	ATP17	GRE3	FMP52	ANB1	PRC1	COX9	MEF2	EMP24	YSA1	YBL029C-A	ATP2	BGL2
YHR182W	PHO3	ATP16	ADD37	ATP3	COX4	GGA1	TPK2	QCR6	AHP1	GRX8	PNP1	YMR315W	FPR2	PYK2	CPA2
COF1	IRC24	SDS24	ETR1	EMI2	SDS22	GIC2	REE1	NAS6	QCR8	LSC1	ALD4	YLR118C	ATP4	PHM8	ATG19
OM14	COX5B	CPR3	RPE1	RMD5	TUB1	HFD1	CDC8	RFA3	CMK2	SPO12	CPR1	ATH1	ECM15	YHI9	YKL091C
SSP120	PHB2	CKB1	COX13	ERV1	BMH2	YER010C	ARG5,6	TPK3	ARI1	MDH1	UGP1	TIM13	MAD2	HSP104	COX5A
CIT1	ERV2	CDC21	YOR111W	GTF1	COX12	IDH1	PUT2	PDB1	HMF1	MAG1	DAP1	GPX2	GVP36	RAV2	YDL086W
YUH1	ATP20	ACO1	ATP7	CIR1	HSP78	TRP1	GDH1	SOD1	ABF2	LDH1	COR1	YIR035C	YMR31	IDH2	CYT2
AIM2	ARG7	CTT1	PHB1	GET3	YKL133C	DDP1	MIR1	ADR1	LSP1	RBK1	RTN2	TUB3	GLO2	PPN1	ARL3
YGR201C	ECM31	HNT1	YBR139W	STF1	FMP10	BOS1	BBC1	MZM1	GDE1	PET54	ATP18	NIT3	IML2	SGT2	PRE7
UBC1	BMH1	PFY1	APE2	PDX3	PEX19	CAB4	CPR5	BOL1	ERP1	GUT2	YFR045W	YIH1	ARG3	ASK10	CAP1
MIC19	DPM1	CPR6	YER134C	UBP15	YMR226C	PAM17	FET5	MTC3	YML079W	XKS1	PRE10	PRE8	MYO4	RFS1	KGD2
YPR148C	THI6	MSW1	PDI1	COX20	PRE5	CMK1	FSH3	ODC1	WTM1	GNA1	YDL066W	SSD1	CDC48	ATG7	IAH1
EXG1	ADO1	FUM1	CMC2	CTS2	AIM45	RRF1	HEM15	YOR131C	ECM29	GFA1	PET191	FPR1	CAP2	UTH1	YMR087W
YBR225W	YFR006W	CDC28	VMA4	RPO21	YPR127W	QCR10	RGC1	YBL036C	DUG1	ARH1	SOL2	PUN1	BER1	RPN10	YPT52
MET14	BUD4	ABZ2	EGD1	CUB1	ATP1	NPL4	VAM7	ESC1	TRX2	ERP4	MET13	VPS29	ARG1	CLC1	GLC7
POS5	LCL2	RDL2	YKL033W-A	GPN2	CCP1	SBP1	UTR4	SBA1	PUP2	NDE1	AIM36	LAG2	YOL157C	HRI1	PUP1
LAP2	KRE5	FAB1	AIM24	EUG1	YKL069W	PPA2	SNF7	ECM14	UBC4	AIM39	TWF1	AIM10	SDH2	RIM15	UFD1
CPS1	PRE9	RSM27	ESS1	ACT1	ECM21	TAE1	FBP26	PRE3	MSS51	YLR179C	ARC15	COQ5	GIM5	YPT31	KAP95
JAC1	YPR172W	RSM19	RTC1	PRS2	YDL144C	EFM4	ARP3	RRD2	UFD4	DAK1	AHA1	SUI1	HSP10	MCM7	OTU1
YAK1	PRM15	ENT3	AAP1	PAA1	MED4	PRE1	DID2	GPM1	YPR1	MDM10	CNE1	PIL1	SNQ2	ZTA1	YMR099C
REX2	YPL225W	TOP2	SNX3	YDR476C	VPS74	RIM11	YGR111W	LAP3	RUP1	UBP2	PHO85	EPO1	BCY1	CTO1	ZIM17
KAP120	YGL185C	COQ11	SCL1	TAL1	LYS1	ERG9	RVS161	ATP11	AIM29	PRD1	PDC6	MIA40	SCW4	LPD1	YOR261C
BAT1	NUM1	LEU1	PAN6	YET3	CRM1	RPN5	HSP60	PCM1	MEF1	TDA10	TAP42	CBS2	TMA20	SSQ1	ARO8
TRP5	GET4	CHC1	GDI1	YIM1	SEC28	FRQ1	RPT4	OXP1	VMA1	EMC5	YAR1	ROT2	CYR1	HSM3	IRR1
CBR1	ASF1	RPT6	CAF16	ISN1	APD1	FMP41	MSD1	ADP1	COX11	YPR156C	DLD2	NTE1	NMA111	STE23
PDC5	SKP1	KIC1	PSK1	YKT6	RPN9	ISD11	AKL1	FAS1	POB3	MPD2	EMP46	CDC10	ADD66	CBP6	MAS2
HEM12	BNA3	DOS2	GUK1	GRS2	CKI1	FAA1	MSP1	MIC60	LEU4	RCK2	PHO86	GIM4	PRE6	SQT1	MTD1
PTC7	ADH6	COQ1	ALO1	OSW7	YGL159W	IMG2	PFD1	YDR381C-A	NIF3	APE3	MGM101	ARO1	COQ8	DOA1	CDC37
YET1	SEC13	HCH1	MUK1	FBA1	AIP1	ERG13	MDH3	GCV1	HOG1	PRE2	UBP6	TRP3	YFR016C	PGK1	YGR130C
SFA1	LAT1	VMA6	TPS3	RSM26	TDH3	DOT5	NAT5	GOS1	HAM1	IPP1	ERG20	RPT5	SFH5	ATG27	RPN12
FAS2	RPN6	NFU1	NAP1	YDR457W	QRI1	ADE16	VPS21	SRV2	TPD3	RAD23	ERG6	PDA1	ACS2	UBA1	ILV5
PIM1	RFA1	TOM40	OSM1	SOL3	RHO2	CYM1	AOS1	SSA1	MRPS16	YKR018C	SMC2	HEM14	RPN1	KGD1	GPD2
RVB2	LYS12	EDE1	EGD2	PGI1	ODC2	CHS5	ALD5	SUB2	SEC14	SEC17	PDC1	SSZ1	TAF10	AAT2	PDR16
ENO2	TMA108	YRB1	YJR045C	RPT1	MOB2	GYP8	YOR133W	PFK2	GND1	SEC62	HRT3	FRS1	VIP1	ABP140	NUP85
TFA1	UPF3	APA1	CYC8	RPS7A	SLA1	RTN1	DLD3	GUA1	ASC1	MSY1	BUD6	GCN20	TYS1	MSH6	UME1
STI1	RPN3	GCV2	VPH1	RGD2	DED81	CSE1	RPB2	SEC63	CBP2	SRP101	NMT1	MET6	GLN3	HSC82	CBF2
YPL220W	DYS1	RPC34	YCR087C-A	SAC1	RBG2	MNN9	MDM20	MCM6	SPE2	FUN30	ADE5,7	LAC1	RAI1	MID2	PMR1
MET12	SHM2	SKI8	HEM1	SST2	UTP13	GTB1	YKR057W	MEX67	PTH2	RPS3	GCD7	GGC1	HIS4	FUN12	NUP188
ABF1	NOP2	TOM70	PRP2	YPP1	RPS25A	GUS1	HGH1	FSH1	RNR1	SFB2	ERG1	POL2	NOP6	RNA14	ARO2
GAS1	ILV3	GLN1	MYO5	KTR3	UTP5	CDC60	CCT7	NUP192	YHB1	SEC12	ARP5	YME1	GRX4	PEX1	HAL9
KTR1	SRP14	HOM3	HPC2	RPG1	YPR102C	RTG2	ARP4	TRM3	TIM50	NIP1	YNL247W	YOR312C	GLT1	MET22	YER117W
STE12	PEP5	LOS1	GRS1	CCT5	CWC22	CCS1	SAP190	RGD1	TPT1	HBS1	SSB2	EAF1	HAL5	YRB2	PRP21
RPS5	CDC9	XRN1	ECM1	RRP7	THO1	MNN2	SPT5	SEC39	CST26	SEC1	MRS6	PGC1	KRS1	CDC73	HIS1
YPL199C	SCP160	KTI12	RPL30	GNT1	VBA4	NCL1	NOT3	GSF2	GAS3	ALA1	SRP21	RNH201	RBG1	EFG1	ADE3
ANT1	PUS4	SKI3	RPL10	ADE13	SWP82	STE20	MCM2	PTC2	RSC6	SUA5	ELP2	SEY1	UTP9	EFM2	MLP2
NUP145	RSR1	TRP4	BYE1	TFB1	YIL018W	SPC42	EMW1	MSI1	YMR1	TCD1	MRN1	UTP18	YGR210C	GAL11	YHR020W
TAF4	ARP7	CRN1	RTT102	CNS1	ERG5	YGL008C	TRM732	IFA38	ARP9	YRA1	UTP20	ERG4	RPS7B	SNU66	RET1
RPL8A	PUS1	ARX1	TRM82	NUP100	STO1	EFR3	MRT4	IES1	GGA2	DIC1	SIR2	RIX7	ECM16	VPS9	TRM7
SNF12	URA4	APM3	ADE6	POL3	YBR242W	DBP10	SEC61	RMD1	PUF6	ELP3	URA7	SKY1	TOR1	HEK2	TAF1
RPL4B	UNG1	STE7	PTA1	LEM3	INO80	TFC1	LEU9	HDA1	RCM1	NEW1	KRR1	GSH1	PAB1	SYF1	CUE3
NOC4	RRP12	UBC6	RPP1A	CTS1	PUS7	NSP1	STE5	PUF3	BUD20	BCH2	RPC53	RPL13B	EAF3	YPR132W	NOP1
GIN4	YNL320W	RCL1	CUS1	NIC96	DBF20	RPL6B	YKL020C	SLS1	PDE2	REX3	VHR1	RPL24B	TYW1	URB2	BRE5
SEC23	ILS1	TUP1	VMS1	RPA34	RPS1B	DIS3	TRM12	TAF12	BCP1	CET1	LOC1	MIT1	DDR48	RPL8B	BOI2
CHD1	RSC8	FPR4	YPL245W	SDO1	PAP2	OGG1	HPR1	UTP10	RPO31	MDL1	RFC1	IOC4	UTP22	PRO1	SAP155
RRN3	MNN11	SUI3	BNA4	URA1	RRB1	POL12	VAS1	RLF2	MGS1	SNU71	SRO9	RPP1	RRP1	SUR2	DPH2
SSA2	ROG1	YVH1	YEF3	LIA1	LTV1	MES1	ISW1	FCF2	POL5	SBE2	NBA1	RPA135	RPL9A	TFG1	LYS4
SEA4	NMD5	MAE1	BSP1	RPS1A	NCS6	CEF1	NOP8	JIP5	ERG11	PAP1	UBP3	VID27	SNF2	BUD27	SAP185
NOP56	ERG3	DBP8	DHH1	BRX1	TRM8	GEF1	MRPL22	NMD2	YHR010W	YCK1	RRP36	VTC4	RNH70	SIS2	NAT1
MSS116	POM34	NAB2	TRM5	TRM1	YCR016W	EPS1	NIS1	UTR2	NPL6	NAM7	RPP2B	RRP9	FUR1	RSC4	RER1
THS1	CDC4	ETT1	PEP3	TPA1	BUD21	ERB1	RPL16B	RAP1	GAR1	CBF5	RPS2	SIR3	SEG1	IPI3	RPL34B
RIX1	YGR054W	SNU13	SYH1	ADE17	RPF1	URB1	DRE2	GDA1	SKO1	RPL7B	RPF2	MNN10	ERJ5	SCT1	BMS1
PAL1	RSA4	TBF1	GIS2	NST1	HXT3	CTK1	EAP1	RPA190	RPL3	RIO1	GYP1	CTR9	RBA50	RPC82	PUF4
UBP8	TRM44	CPA1	GCD10	DBP3	YDR225W	DPH1	UTP7	MTR4	BUL2	DBP6	TAH18	MIS1	KRE33	ENP2	TIF3
SBE22	ECM25	YHM2	MRD1	ENB1	DUS3	SOF1	PRP43	BIO2	TOP1	LAA1	DIP2	RQC1	PIS1	TSR1	BFR2
SIN3	NRD1	YMR265C	SKP2	RAD30	DBP2	SKN7	MIG2	SPL2	VPS34	RIF1	RRP17	YPL090C	SUP35	IPI1	RDH54
DBP7	IDP2	YOR182C	NUP60	SCS7	NUP57	UTP11	TEX1	FCY2	NAN1	MPP10	UTP30	SMY2	ESF2	PBP1	ALB1
YER102W	SRP68	YHR056C	NOP12	SGD1	GLN4	MNN5	UBP10	PRP11	RRP45	NOP9	ENP1	STH1	RSC2	NOP58	DBP9
UTP6	LRS4	PRP28	DED1	PRP6	YKR094C	CDC14	PSA1	MOB1	MAK5	LCP5	VTC2	TMA16	LSG1	SPT2	PWP2
RPA49	CIC1	SPB1	GLO3	TIF4631	REI1	HRR25	MUD1	RTT107	PXR1	MSA2	BEM3	SRP72	AAH1	MRH1	RAD5
MDG1	BUD22	RPL33A	POP1	GCD2	SET3	HXT2	ITR1	AIM21	RBD2	YPR043W	YBL002W	RLI1	RPL5	ORC4	RKI1
YPF1	UTP25	NOP15	BUL1	YNL217W	ORC2	YNL162W	ELO3	HEL2	QDR2	YOL130W	BDF1	TGS1	RPL16A	SRP40	NOP16
SRP54	OLE1	SPC110	GLE1	UTP4	DRN1	EBP2	TRL1	IES2	CIT2	NUG1	MAK21	NUP53	NUP2	NOG1	YOL120C
RSC58	PRP19	NRP1	SAS10	YCK2	TEF4	RTF1	SAT4	TRM2	VTC3	RPL37B	RMT2	YNL030W	ARB1	PPT1	COG3
SSF2	RXT3	SPE1	EDC1	NOC2	NOP13	YJU2	THP3	GCD11	CTR1	ROK1	MOT1	SEN34	RRP8	DUS1	MSL5
UTP8	NSA2	DIM1	FKH2	RRT14	UTP14	KRI1	SGV1	TCB2	TSC10	VTC1	NOB1	YBL081W	IXR1	UTP23	PAT1
ARO10	NOP7	AIR1	YDR025W	NOP53	TMA46	PNO1	SCY1	NOC3	SPP41	RLP24	HTZ1	NOP14	GDS1	PHO90	RSC3
MNN1	HAS1	DOT6	HCA4	ESF1	BUD23	SPB4	RSC1	MED6	NHP6A	PCT1	YNL050C	SLX9	PEF1	FAF1	VTS1
NOP4	MKT1	RPS26B	CDC1	RRP6	REB1	YMC2	GNP1	HHO1	ULS1	RLP7	RSC9	FAA3	RRP14	NOG2	YGR026W
EKI1	PHO84	RSN1	DRS1	FAP1	YBR238C	PHO4	YBL028C	ATF2	SDA1	NUP49	DIP5	BRE1	PEX12	NDC80	TAT1
SET2	LYP1	VHR2	STE3	YPR114W	BAP2	ATF1	YGL101W	YPK3	CHA1	ENV10	LYS14	HST1	PUS9	PHO89	GZF3
MLS1	DYN1	YNL031C	ERG24	YMR310C											

log2 (fold change)

5.0

2.5

0.0

- 2.5

- 5.0
